# Supplementary material for: Childhood conditions, pathways to entertainment work and current practices of female entertainment workers in Cambodia: Baseline findings from the Mobile Link trial
Source: PLoS One. 2019 Oct 15;14(10):e0216578. doi: 10.1371/journal.pone.0216578 (PMC6793882; doi:10.1371/journal.pone.0216578)
Supplement: S1 File — (PDF) [file pone.0216578.s001.pdf]

Supporting Information 1: Baseline Questionnaire in English

Questionnaire

Survey Information:

Variables Name	Response	Code
Study sites	Phnom Penh<br>Battambang<br>Banteay Meanchey<br>Siem Reap	01<br>02<br>03<br>04
Type of study participant	Intervention<br>Control	01<br>02
Unique ID code	Study site code + Location + serial 001	Eg. 0101001
Date of interview		
Interviewer name	Am Sa Em<br>Chan Sreymom<br>Leng Sokny<br>Lmot Dina<br>Neur Chanda<br>Hieng Menglang<br>Ra Romphoun<br>Chhay Thida<br>Ann Dariya<br>Heang Mouyim	01<br>02<br>03<br>04<br>05<br>06<br>07<br>08<br>09<br>10
QC name		

Question ID	Questions	Response	Response Code	Remark
**Section 1: Demographics and background**				
1.	How old are you at your last birthday?	.... years		
2.	In what province were you born?	BANTEAY MEANCHEY<br>BATTAMBANG<br>KAMPONG CHAM<br>KAMPONG CHHNANG<br>KAMPONG SPEU<br>KAMPONG THOM<br>KAMPOT<br>KANDAL<br>KOH KONG<br>KRATIE<br>MONDUL KIRI<br>PHNOM PENH<br>PREAH VIHEAR<br>PREY VENG<br>PURSAT<br>RATANAK KIRI<br>SIEM REAP<br>KRONG PREAH SIHANOUK<br>STOEUNG TRENG<br>SVAY RIENG<br>TAKEO<br>OTDAR MEANCHEY<br>KRONG KEP<br>KRONG PAILIN<br>Tboung Khmum<br>Abroad	1<br>2<br>3<br>4<br>5<br>6<br>7<br>8<br>9<br>10<br>11<br>12<br>13<br>14<br>15<br>16<br>17<br>18<br>19<br>20<br>21<br>22<br>23<br>24<br>25<br>26	
3.	In what type of community were you born?	Rural<br>Urban	1<br>2	
4.	Are your parents both still alive?	Both are death<br>Both still alive<br>Mother or Father still alive<br>Don't know	0<br>1<br>2<br>3	
5.	Thinking of your childhood home, what type of roofing did you have?	Wood planks<br>Plastic sheet<br>Iron/Aluminum<br>Wood<br>Ceramic tiles	1<br>2<br>3<br>4<br>5	

		Cement<br>Concrete<br>Thatch/Leaves<br>Don't know<br>Other:_________________	6<br>7<br>8<br>9<br>10	
6.	Thinking of your childhood home, did you have piped water into your home?	No<br>Yes<br>Not Sure	0<br>1<br>2	
7.	Thinking of your childhood home, did you have electricity in your home?	No<br>Yes<br>Not sure	0<br>1<br>2	
8.	Thinking of your childhood home, what was the main material of the floor?	Wood /Bamboo planks<br>Plastic sheet<br>Clay<br>Ceramic tiles<br>Cement<br>Stone<br>Don't know<br>Other:_________________	1<br>2<br>3<br>4<br>5<br>6<br>7<br>8	
9.	When you were a child, did you often not have enough food?	No<br>Yes<br>Not sure	0<br>1<br>2	
10.	When you were a child, could your family afford to send you to school?	No<br>Yes	0<br>1	
11.	How many years of schooling have you completed?	………………..years<br>Note: 0 if never attended		
12.	What is your current marital status?	Married and living together<br>Married, but not living together<br>Widowed/divorced/separated<br>Never married, not living with a sexual partner<br>Never married, but living with a sexual partner	1<br>2<br>3<br>4<br>5	
13.	What type of house are you currently living in?	My own/ family house<br>Rental house on my own<br>Rental house with my family<br>Rental house shared with friends<br>Dormitory at my work place<br>Homeless (on the street or public premise)<br>Other:_________________	1<br>2<br>3<br>4<br>5<br>6<br>7	
14.	How many children do you have?	………………….. Children		
15.	Whom are you currently living with?	Boyfriend/sweetheart<br>Husband<br>Family (parents, siblings, children)<br>Relatives<br>Friends/colleagues	1<br>2<br>3<br>4<br>5	

		Other:________________	6	
16.	How many people are dependent on you for living?	……….. people		
17.	Are there anyone else contributing to support your family?<br>(multiple answer)	None<br>Boyfriend/sweetheart<br>Husband<br>Family (parents, siblings, children)<br>Relatives<br>Other:________________	0<br>1<br>2<br>3<br>4<br>5	
**Section 2: Entertainment work**				
18.	For how long have you been living in the current city?	………..Years<br>0 if not living in this city<br>1 if living in this city <1 year		
19.	Have you moved from another city to this city?	No<br>Yes	0<br>1	0 Skip to Q21
20.	What was the top reason you moved to this city?	For economic opportunities<br>Following family members<br>Interest in new places<br>Getting away from a bad situation<br>Other:________________	1<br>2<br>3<br>4<br>5	
21.	Have you ever worked in the garment industry?	No<br>Yes	0<br>1	0 Skip to Q23
22.	What was the main reason for your leave the garment industry?<br>(multiple answer)	Better pay elsewhere<br>Better working conditions elsewhere<br>Was fired or laid off<br>Interested in a different job<br>Offered another job<br>Other:________________	1<br>2<br>3<br>4<br>5<br>6	
23.	How many months ago did you start working in entertainment industry?	……………………..months<br>Note: 1 if less than 1 month		
24.	What type of venue best describes your first job in entertainment?	Karaoke bar<br>Massage parlor<br>Beer garden<br>Restaurant/cafe<br>Dance club<br>Freelance (street, public parks)<br>Other:________________	1<br>2<br>3<br>4<br>5<br>6<br>7	
25.	What type of venue best describes your current job in entertainment?	Karaoke bar<br>Massage parlor<br>Beer garden<br>Restaurant/cafe<br>Dance club<br>Freelance (street, public parks)<br>Other:________________	1<br>2<br>3<br>4<br>5<br>6<br>7	

26.	How much money do you typically make per month at your current entertainment job?	……………………. US$		
27.	Are you a part of any organizations that support entertainment/sex workers?	No<br>Yes	0<br>1	
**Section 3: Sexual behaviors**				
28.	Have you had sexual intercourse in the past 3 months?	No<br>Yes	0<br>1	0 skip to Q44
29.	In the past 3 months, did you have sexual intercourse with a partner not in exchange for money or gift (boyfriend/sweetheart)?	No<br>Yes	0<br>1	0 skip to Q34
30.	In the past 3 months, with how many partners did you have sexual intercourse not in exchange for money or gift?	……………partner(s)		
31.	The last time you had sexual intercourse with a partner not in exchange for money or gift, did your partner use a condom?	No<br>Yes	0<br>1	
32.	In the past three months when you had sexual intercourse with a partner not in exchange for money or gifts, how often did you use a condom?	Always<br>Frequently<br>Sometimes<br>Never	1<br>2<br>3<br>4	
33.	The last time when you had sexual intercourse without a condom with a partner not in exchange for money or gifts, what was the main reason for not using it?	Trust partner<br>Condom was not available<br>I requested but my partner did not want to use condom<br>I requested but my partner convinced me that it was ok<br>I did not request because I felt uncomfortable or fearful asking<br>I did not request because I did not think about it<br>I did not like using condoms<br>I'm scared that using a condom might have a negative effect on my body<br>Other:_________________	0<br>1<br>2<br>3<br>4<br>5<br>6<br>7<br>8	
34.	In the past 3 months, did you have sexual intercourse with a partner in exchange for money or gift (clients)?	No<br>Yes	0<br>1	0 skip to Q44

35.	What was the main reason for your involvement in sex work?<br>(Multiple answer possible)	In need of money<br>Suggested by a friend<br>Lured, cheated or forced into it<br>To get out of bad family situation<br>To get out of other work you did not like<br>Other:_________________	1<br>2<br>3<br>4<br>5<br><br>6	
36.	How old were you the first time you had sexual intercourse with a partner in exchange for money or gift?	……………………. years		
37.	In the past 3 months, how often did you have sexual intercourse with a partner in exchange for money or gifts?	Daily<br>A few times a week<br>Weekly<br>Monthly<br>Once in a while when I want or need to	1<br>2<br>3<br>4<br>5	
38.	Where do you usually meet these types of partners (clients)?	At work<br>Through friends<br>At social gatherings<br>On the street<br>Advertisement/phone call<br>Other:_________________	1<br>2<br>3<br>4<br>5<br>6	
39.	In the past 3 months, with how many partners did you have sexual intercourse in exchange for money or gift?	……………..partner(s)		
40.	In the past 7 days, with how many partners did you have sexual intercourse in exchange for money or gift?	……………..partner(s)		
41.	The last time you had sexual intercourse with a partner in exchange for money or gift, did your partner use a condom?	No<br>Yes	0<br>1	
42.	In the last 3 months when you had sexual intercourse with a partner in exchange for money or gift, how often did your partners use a condom?	Always<br>Frequently<br>Sometimes<br>Never	1<br>2<br>3<br>4	
43.	The last time when you had sexual intercourse without a condom with a partner in exchange for money or gift, what was the main reason for not using it?	Trust partner<br>Condom was not available<br>I requested but my partner did not want to use condom<br>I requested but my partner convinced me that it was ok<br>I did not request because I felt uncomfortable or fearful asking	0<br>1<br>2<br><br>3<br><br>4<br><br>5	

		I did not request because I did not think about it<br>I did not like using condoms<br>I'm scared that using a condom might have a negative effect on my body<br>Other:________________	6<br>7<br>8	
**Section 4: Condom use self-efficacy**				
44.	Can you discuss condom use with any sexual partner you might have?	Definitely no<br>Sometimes<br>Definitely yes	0<br>1<br>2	
45.	Can you insist on condom use whenever you are with someone who says they do not have any disease?	Definitely no<br>Sometimes<br>Definitely yes	0<br>1<br>2	
46.	Can you suggest using a condom when you do not initiate the sexual activity?	Definitely no<br>Sometimes<br>Definitely yes	0<br>1<br>2	
47.	Can you insist on condom use if a partner does not want to use one?	Definitely no<br>Sometimes<br>Definitely yes	0<br>1<br>2	
48.	Can you stop a sexual encounter if a partner insists on not using a condom?	Definitely no<br>Sometimes<br>Definitely yes	0<br>1<br>2	
49.	Can you continue to insist on condom use with a person who gets angry when you suggest it?	Definitely no<br>Sometimes<br>Definitely yes	0<br>1<br>2	
**Section 5: HIV risk perception, testing and treatment**				
50.	Do you feel that you are at risk for HIV?	No<br>Yes<br>Not sure	0<br>1<br>2	0 Skip to Q52
51.	IF Yes, why do you feel you are at risk for HIV?<br><br>(check all that apply)	I know my partner(s) is HIV +<br>I frequently have unprotected sex<br>I do not trust my husband/partner<br>I shared needles to inject drugs<br>I have family members who are HIV+<br>I'm a caretaker for an HIV+ individual<br>Other____________	1<br>2<br>3<br>4<br>5<br>6<br>7	
52.	If No, why don't you feel you are at risk for HIV?<br><br>(Check all that apply)	Never had sex/ no partner<br>I only have sex with my husband<br>I don't think my partners are HIV+<br>I don't feel sick<br>I always use condoms<br>I get HIV testing every 3-6 months	0<br>1<br>2<br>3<br>4<br>5	

		I wash after having sex every time<br>I get cleaned at a clinic frequently<br>Other__________________	6<br>7<br>8	
53.	Have you ever been tested for HIV test?	No<br>Yes	0<br>1	0 skip to Q61
54.	In the past 6 months, have you been tested for HIV?	No<br>Yes	0<br>1	0 skip to Q61
55.	Where did you have your most recent HIV test?	Private facilities<br>Public facilities<br>NGO facilities<br>NGO outreach workers at workplace or home<br>Community outreach of NGO<br>Other__________________	1<br>2<br>3<br>4<br>5<br>6	
56.	If you don't mind, could you please let us know your HIV status?<br><br>Note: You can choose not to answer	HIV(-)<br>HIV(+)<br>I don't know<br>Don't want to answer	1<br>2<br>3<br>4	1 skip to Q61<br><br>4 skip to Q61
57.	Are you currently on ART?	No<br>Yes<br>I was but discontinued<br>I am on pre-ART	0<br>1<br>2<br>3	0 2 3 skip to Q60
58.	Do you take ARV regularly as prescribed?	Not regularly<br>Regularly	0<br>1	
59.	Where do you get ART services?	Public facilities<br>Private facilities<br>NGO facilities<br>Pharmacy<br>Other_________________	1<br>2<br>3<br>4<br>5	All skip to Q61
60.	If not, why are you not on ART?	Not needed<br>Not sure where to go<br>Ashamed to go to health facility<br>Afraid of being discriminated<br>Badly treated in the past<br>Other_________________	1<br>2<br>3<br>4<br>5<br>6	
**Section 6: STI testing and treatment**				
61.	In the past 3 months, have you experienced the following symptoms?<br><br>[Multiple answers]	No symptoms<br>Cuts or sores in the genital area<br>Swelling in the genital area<br>Abnormal urethral discharge<br>Symptom in the mouth/throat<br>Other_________________	0<br>1<br>2<br>3<br>4<br>5	0 skip to Q66

62.	Did you seek screening for STI when you had the most recent symptom?	No<br>Yes	0<br>1	0 skip to Q66
63.	In the past three months, have you been told by a health care provider that you had an STI?	No<br>Yes	0<br>1	0 skip to Q66
64.	Did you receive treatment for your most recent STI?	No<br>Yes	0<br>1	0 skip to Q66
65.	Where did you receive the treatment for the most recent STI?<br><br>(Only one response)	Pharmacy<br>Private clinic/hospital<br>Public clinic/hospital<br>NGO Clinic/hospital<br>Traditional healer<br>Other________________	0<br>1<br>2<br>3<br>4<br>5	
**Section 7: Contraceptive and pregnancy**				
66.	Are you currently using a modern contraceptive to prevent pregnancy?	No<br>Yes	0<br>1	0 skip to Q68
67.	If yes, what types of contraceptive are you using (check all that apply)?	Condom<br>Pill<br>Injectable<br>Intra-uterus devices (IUD)<br>Implant (use under the skin)<br>Female sterilization<br>Male sterilization<br>Other________________	1<br>2<br>3<br>4<br>5<br>6<br>7<br>8	All skip to Q69
68.	If not, why are you not currently using any modern contraceptive method?	Do not like side effects<br>Do not think I need contraception<br>Do not want to prevent pregnancy/want to get pregnant<br>Do not like using modern methods<br>Do not know where to get contraception<br>Do not think I can afford contraception<br>Other________________	1<br>2<br>3<br><br>4<br>5<br><br>6<br><br>7	
69.	Do you usually douche yourself before or after sex as a way to prevent sexually transmitted disease or HIV?	No<br>Yes	0<br>1	0 Skip to Q72
70.	If yes, how often do you douche yourself after sex as a way to prevent sexually transmitted disease or HIV?	Always<br>Frequently<br>Sometimes<br>Never	1<br>2<br>3<br>4	
71.	If you wash after sex, what are the reasons that you wash ?<br>(multiple answer)	Preventing STI<br>Preventing HIV<br>Preventing pregnancy<br>Stay clean	1<br>2<br>3<br>4	

		Avoid smell<br>Other…………………	5<br>6	
72.	Do you think medical abortion (before 12 weeks gestation) is legal in Cambodia?	No<br>Yes<br>Don't know	0<br>1<br>2	
73.	Have you ever experienced unwanted pregnancy?	No<br>Yes<br>Don't know	0<br>1<br>2	0 skip to Q82
74.	Have you ever experienced unwanted pregnancy in the past 12 months?	No<br>Yes<br>Don't know	0<br>1<br>2	
75.	Have you ever had an abortion?	No<br>Yes<br>Don't know	0<br>1<br>2	0 skip to Q82
76.	During your lifetime, how many abortions have you had?	………………. times		
77.	In the past 12 months, how many abortions have you had?	………………. times		
78.	The last time you had an abortion, where did you get services?	Pharmacy<br>Private clinic/hospital<br>Public clinic/hospital<br>NGO Clinic/hospital<br>Traditional healer<br>Other_________________	0<br>1<br>2<br>3<br>4<br>5	
79.	Did you experience any complication from your most recent abortion such as excessive bleeding or infection?	No<br>Yes	0<br>1	0 skip to Q82
80.	If yes, did you seek treatment for these complications?	No<br>Yes	0<br>1	0 skip to Q82
81.	If yes, where did you seek the treatment?<br>(multiple answer)	Pharmacy<br>Private clinic/hospital<br>Public clinic/hospital<br>NGO Clinic/hospital<br>Traditional healer<br>Other_________________	0<br>1<br>2<br>3<br>4<br>5	
**Section 8: Gender-based violence**				
82.	Have you experienced unwanted touching or groping in the past 3 months? (at work?)	No<br>Yes	0<br>1	
83.	What can you do if you or a female friend or family member experience physical or sexual abuse?<br>(multiple answer)	There is nothing to do<br>Go to local authorities<br>Go to police or courts<br>Tell other family and friends<br>Go to an NGO<br>Other_________________	1<br>2<br>3<br>4<br>5<br>6	

84.	If a wife does not obey a husband, do you think he is justified in hitting, kicking or beating her?	No<br>Yes<br>Sometimes	0<br>1<br>2	
85.	If a wife does not obey a husband, do you think he is justified in yelling at her?	No<br>Yes<br>Sometimes	0<br>1<br>2	
86.	If a girlfriend does not obey a boyfriend, do you think he is justified in hitting, kicking or beating her?	No<br>Yes<br>Sometimes	0<br>1<br>2	
87.	Do you think it is a woman's duty to have sex with her husband, even when she does not want to?	No<br>Yes<br>Sometimes	0<br>1<br>2	
88.	What type of violence, if any, have you ever experienced in your lifetime?<br><br>(multiple answer)	None<br>Verbal threats<br>Controlling ability to leave house<br>Physical abuse<br>Forced sex<br>Forced to use alcohol<br>Forced to use drug<br>Other_________________	0<br>1<br>2<br>3<br>4<br>5<br>6<br>7	0 skip to Q91
89.	What type of violence, if any, have you experienced in the past six months?<br><br>(check all that apply)	None<br>Verbal threats<br>Controlling ability to leave house<br>Physical abuse<br>Forced sex<br>Forced to use alcohol<br>Forced to use drug<br>Other_________________	1<br>2<br>3<br>4<br>5<br>6<br>7<br>8	0 skip to Q91
90.	Who was the main perpetrator of the violence?	Family member<br>Friend<br>Husband/Partner<br>Sweetheart<br>Manager<br>Client<br>Co-worker<br>Stranger<br>Local authority/police<br>Moto/taxi driver<br>Other_________________	1<br>2<br>3<br>4<br>5<br>6<br>7<br>8<br>9<br>10<br>11	
**Section 9: Substance abuse**				
91.	In the past 3 months, how often did you drink at least one can of beer or one glass of any types of wine?	Never<br>Once a month or less<br>2-4 times a month<br>2-3 times a week<br>4 or more times a week	 0<br>1<br>2<br>3	0 skip to Q94

	- A glass of wine (120 ml)<br>- A glass of whisky (30 ml)		4	
92.	In the past 3 months, how many standard drinks containing alcohol (a can of beer or a glass of any types of wine) did you have on a typical day on which you drank alcohol?	1 - 2<br>3 – 4<br>5 - 6<br>7 - 9<br>10 or more	0<br>1<br>2<br>3<br>4	
93.	In the past 3 months, how often did you have more than 4 drinks in 24 hour ?	Never<br>Less than once a month<br>Once a month<br>1, 2, or 3 times a week<br>4 or more times a week	0<br>1<br>2<br>3<br>4	
94.	In the past 3 months, how often have you been forced to drink alcohol at work when you did not want to drink?	Never<br>Less than once a month<br>Once a month<br>1, 2, or 3 times a week<br>4 or more times a week	0<br>1<br>2<br>3<br>4	
95.	In the past 3 months, have you used any of the following drugs?<br>(Ask one by one – CIRCLE YES OR NO)	Never<br>Marijuana<br>Heroin/opium<br>Yama (amphetamine)<br>Crystal, Ice (methamphetamine)<br>Ecstasy<br>Inhalants (glue, paint, petrol, spray can)<br>Other ………… (specify)	0<br>1<br>2<br>3<br>4<br>5<br>6<br>7	0 skip to Q98
96.	In the past 3 months, have you injected any illicit drugs?	Never injected any drug<br>Heroin<br>Yama<br>Crystal, Ice<br>Other_________________	0<br>1<br>2<br>3<br>4	
97.	In the past 3 months, have you had sex during or after using illicit drugs?	No<br>Yes<br>Don't want to answer	0<br>1<br>2	
**Section 10: Linkage to services**				
98.	Have you ever contacted an outreach worker to ask a health question in the past 6 months?	No<br>Yes	0<br>1	0 end of intervie w

99.	What health issues did you contact her about?<br><br>(check all that apply)	HIV<br>STIs<br>Family planning<br>Gender-based violence<br>General emotional support<br>Vaginal health (discharge, irritation, inflammation)<br>Legal support<br>Other________________	1<br>2<br>3<br>4<br>5<br>6<br><br>7<br>8	
100.	In the past 6 months, how many times have you contacted an outreach worker?	Never<br>1 time<br>2-4 times<br>5+ times	0<br>1<br>2<br>3	
101.	Have you ever received an escorted referral for a health services from an outreach worker?	No<br>Yes	0<br>1	0 end of interview
102.	For what health issues did you receive an escorted referral?<br><br>(check all that apply)	HIV<br>STIs<br>Family planning<br>Gender-based violence<br>General emotional support<br>Vaginal health (discharge, irritation, inflammation)<br>Legal support<br>Other________________	1<br>2<br>3<br>4<br>5<br>6<br><br>7<br>8	

Thank you for your participation!
