# Supplementary material for: Childhood conditions, pathways to entertainment work and current practices of female entertainment workers in Cambodia: Baseline findings from the Mobile Link trial
Source: PLoS One. 2019 Oct 15;14(10):e0216578. doi: 10.1371/journal.pone.0216578 (PMC6793882; doi:10.1371/journal.pone.0216578)
Supplement: S2 File — (PDF) [file pone.0216578.s002.pdf]

**Supporting Information 2: Baseline Questionnaire in Khmer**

# កម្រងសំណួរ

<u>ពត៌មានទូទៅ៖</u>

| អថេរ | ចម្លើយ | កូត |
|---|---|---|
| ទីតាំងនៃការសិក្សា | ភ្នំពេញ<br>បាត់ដំបង<br>បន្ទាយមានជ័យ<br>សៀមរាប | 01<br>02<br>03<br>04 |
| ប្រភេទអ្នកចូលរួម | ក្រុមអន្តរាគមន៍<br>ក្រុមប្រៀបធៀប | 01<br>02 |
| លេខកូដអ្នកចូលរួម | កូដទីតាំង+លេខកូដOD+serial 001<br>ឧទា. 0101001 | |
| កាលបរិច្ឆេទសម្ភាសន៍ | ថ្ងៃ........ខែ.........ឆ្នាំ........... | |
| ឈ្មោះអ្នកសម្ភាសន៍ | Am Sa Em<br>Chan Sreymom<br>Leng Sokny<br>Lmot Dina<br>Neur Chanda<br>Hieng Menglang<br>Ra Romphoun<br>Chhay Thida<br>Ann Dariya<br>Heang Mouyim | 01<br>02<br>03<br>04<br>05<br>06<br>07<br>08<br>09<br>10 |
| ឈ្មោះអ្នកត្រួតពិនិត្យគុណភាព | | |

| លេខកូដសំណួរ | សំណួរ | ចម្លើយ | លេខកូដ | សម្គាល់ |
|---|---|---|---|---|
| **ផ្នែកទី ១៖ ប្រជាសាស្ត្រ និងសារតាទូទៅ** | | | | |
| 1 | តើអ្នកអាយុប៉ុន្មានគិតត្រឹមថ្ងៃកំណើតចុងក្រោយរបស់អ្នក? | --------------- គិតជាឆ្នាំពេញ | | |
| 2 | តើអ្នកកើតនៅខេត្តណា? | បន្ទាយមានជ័យ<br>បាត់ដំបង<br>កំពង់ចាម<br>កំពង់ឆ្នាំង<br>កំពង់ស្ពឺ<br>កំពង់ធំ<br>កំពត<br>កណ្តាល<br>កោះកុង<br>ក្រចេះ<br>មណ្ឌលគិរី<br>ភ្នំពេញ<br>ព្រះវិហារ<br>ព្រៃវែង<br>ពោធិសាត់<br>រតនៈគិរី<br>សៀមរាប<br>ព្រះសីហនុ<br>ស្ទឹងត្រែង<br>ស្វាយរៀង<br>តាកែវ<br>ឧត្តរមានជ័យ<br>កែប<br>ប៉ៃលិន<br>ត្បូងឃ្មុំ<br>ក្រៅប្រទេស | 1<br>2<br>3<br>4<br>5<br>6<br>7<br>8<br>9<br>10<br>11<br>12<br>13<br>14<br>15<br>16<br>17<br>18<br>19<br>20<br>21<br>22<br>23<br>24<br>25<br>26 | |
| 3 | តើអ្នកកើតនៅក្នុងតំបន់ប្រភេទណា? | ជនបទ<br>ទីប្រជុំជន | 1<br>2 | |
| 4 | តើឪពុកម្តាយរបស់អ្នកនៅរស់ដែររឺទេ? | ស្លាប់ទាំងពីរ<br>នៅរស់ទាំងពីរ<br>នៅរស់ម្តាយ/ឪពុក<br>មិនដឹង | 0<br>1<br>2<br>3 | |
| 5 | គិតអំពីផ្ទះដែលអ្នករស់នៅកាលពីកុមារភាព<br>តើផ្ទះរបស់អ្នកមានប្រភេទដំបូលជាអ្វី? | ក្តារបន្ទះឈើ<br>ជ័រ/ប្លាស្ទិច<br>ដែក/អាលុយមីញ៉ូម<br>ឈើ<br>ក្បឿងសេរ៉ាមិច | 1<br>2<br>3<br>4<br>5 | |

| | | | | |
|---|---|---|---|---|
| | | ស៊ីម៉ង់<br>បេតុង<br>ស្បូវ/ស្លឹក<br>មិនច្បាស់<br>ផ្សេងៗសូមបញ្ជាក់------------------ | 6<br>7<br>8<br>9<br>10 | |
| 6 | គិតអំពីផ្ទះដែលអ្នករស់នៅកាលពីកុមារភាព<br>តើផ្ទះរបស់អ្នកមានប្រព័ន្ធទឹកស្អាតដែររឺទេ? | ទេ<br>ចាស់<br>មិនច្បាស់ | 0<br>1<br>2 | |
| 7 | គិតអំពីផ្ទះដែលអ្នករស់នៅកាលពីកុមារភាព<br>តើផ្ទះរបស់អ្នកមានអគ្គិសនីប្រើប្រាស់ដែររឺទេ? | ទេ<br>ចាស់<br>មិនច្បាស់ | 0<br>1<br>2 | |
| 8 | គិតអំពីផ្ទះដែលអ្នករស់នៅកាលពីកុមារភាព<br>តើផ្ទះរបស់អ្នកក្រាលដោយអ្វី? | បន្ទះក្តារ/បន្ទះឬស្សី<br>ជ័រ/ប្លាស្ទិច<br>ដីឥដ្ឋ<br>សេរ៉ាមិច<br>ស៊ីម៉ង់<br>ថ្ម<br>មិនច្បាស់<br>ផ្សេងៗសូមបញ្ជាក់--------------- | 1<br>2<br>3<br>4<br>5<br>6<br>7<br>8 | |
| 9 | ពេលដែលអ្នកនៅក្មេង<br>តើអ្នកមិនមានអាហារបរិភោគគ្រប់គ្រាន់ញឹកញាប់ដែររឺទេ? | ទេ<br>ចាស់<br>មិនច្បាស់ | 0<br>1<br>2 | |
| 10 | ពេលដែលអ្នកនៅក្មេង<br>តើគ្រួសាររបស់អ្នកមានលទ្ធភាពបញ្ជូនអ្នកទៅសាលារៀនដែររឺទេ? | ទេ<br>ចាស | 0<br>1 | |
| 11 | តើអ្នកបានរៀនប៉ុន្មានឆ្នាំដែរ? | ....................ឆ្នាំ<br>**ចំណាំ:**<br>ដាក់លេខ**0**ប្រសិនបើមិនធ្លាប់រៀន | | |
| 12 | សូមប្រាប់ពីស្ថានភាពអាពាហ៍ពិពាហ៍របស់អ្នក? | រៀបការរស់នៅជាមួយគ្នា<br>រៀបការតែមិនរស់នៅជាមួយគ្នា<br>មេម៉ាយ/ពោះម៉ាយ/បែកគ្នា<br>មិនដែលរៀបការមិនរស់នៅជាមួយដៃគូ<br>មិនដែលរៀបការតែកំពុងរស់នៅជាមួយដៃគូ | 1<br>2<br>3<br>4<br>5 | |
| 13 | តើអ្នកកំពុងតែស្នាក់នៅក្នុងផ្ទះប្រភេទណា? | ផ្ទះផ្ទាល់ខ្លួន/គ្រួសារ<br>ផ្ទះជួលផ្ទាល់ខ្លួន<br>ផ្ទះជួលជាមួយគ្រួសារ<br>ផ្ទះជួលជាមួយមិត្តភក្តិ | 1<br>2<br>3<br>4 | |

| | | | | |
|---|---|---|---|---|
| | | ផ្ទះស្នាក់នៅកន្លែងការងារ<br>នៅតាមដងផ្លូវ/គ្មានផ្ទះសំបែង<br>ផ្សេងៗសូមបញ្ជាក់---------------- | 5<br>6<br>7 | |
| 14 | តើអ្នកមានកូនប៉ុន្មាននាក់? | -------------------នាក់ | | |
| 15 | តើបច្ចុប្បន្ននេះអ្នករស់នៅជាមួយអ្នកណា? | មិត្តប្រុស/សង្សារ<br>ប្តី<br>គ្រួសារ( ឪពុកម្តាយ បងប្អូន កូន)<br>សាច់ញាតិ<br>មិត្តភក្តិ/មិត្តរួមការងារ<br>ផ្សេងៗសូមបញ្ជាក់---------------- | 1<br>2<br>3<br>4<br>5<br>6 | |
| 16 | តើមនុស្សប៉ុន្មានអ្នកដែលរស់នៅពឹងផ្អែកលើចំណូលរបស់អ្នក? | …………នាក់ | | |
| 17 | តើមានអ្នកណាផ្សេងទៀតក្រៅពីអ្នកជួយទំនុកបំរុងគ្រួសារអ្នករឺទេ? (ចម្លើយអាច លើសពីមួយ) | មិនមាន<br>មិត្តប្រុស/សង្សារ<br>ប្តី<br>គ្រួសារ( ឪពុកម្តាយ បងប្អូន កូន)<br>សាច់ញាតិ<br>ផ្សេងៗសូមបញ្ជាក់------------------ | 0<br>1<br>2<br>3<br>4<br>5 | |
| **ផ្នែកទី២៖ ការងារក្នុងកន្លែងកម្សាន្ត** | | | | |
| 18 | តើអ្នករស់នៅក្នុងទីក្រុងនេះបានរយៈពេលប៉ុន្មានហើយ? | ………….ឆ្នាំ<br>**ចំណាំ:**<br>ដាក់លេខ**០**ប្រសិនបើមិនរស់នៅក្នុងទីក្រុងនេះ<br>ដាក់លេខ**១**ប្រសិនបើរស់នៅក្នុងទីក្រុងនេះក្រោម១ឆ្នាំ | | |
| 19 | តើអ្នកផ្លាស់មកពីទីក្រុងផ្សេងមកទីនេះមែនទេ? | ទេ<br>ចាស | 0<br>1 | 0 សូមរំលងទៅសំណួរលេខ21 |
| 20 | តើមូលហេតុចំបងអ្វីដែលធ្វើឲ្យអ្នកផ្លាស់មករស់នៅទីនេះ? | សម្រាប់ឱកាសការងារ<br>មកតាមគ្រួសារ<br>ចាប់អារម្មណ៍កន្លែងថ្មី<br>រត់គេចពីស្ថានភាពមិនអំណោយផល<br>ផ្សេងៗសូមបញ្ជាក់--------------- | 1<br>2<br>3<br>4<br>5 | |
| 21 | តើអ្នកធ្លាប់ធ្វើការនៅរោងចក្រកាត់ដេរដែររឺទេ? | ទេ<br>ចាស | 0<br>1 | 0 សូមរំលងទៅសំណួរលេខ23 |

| | | | | |
|---|---|---|---|---|
| 22 | អ្វីជាមូលហេតុចម្បងបានជាអ្នកឈប់ពីការងាររោងចក្រ?<br>(ចម្លើយមានលើសពីមួយ) | ដើម្បីទៅរកចំណូលប្រសើរឡើងនៅកន្លែងផ្សេងទៀត<br>ដើម្បីរកកន្លែងការងារផ្សេងដែលមានលក្ខខណ្ឌប្រសើរជាង<br>ត្រូវបានគេបញ្ឈប់/ផ្អាកការងារ<br>ចាប់អារម្មណ៍នឹងការងារផ្សេង<br>ត្រូវបានគេផ្តល់ការងារថ្មី<br>ផ្សេងៗសូមបញ្ជាក់--------------- | 1<br>2<br>3<br>4<br>5<br>6 | |
| 23 | តើប៉ុន្មានខែហើយដែលអ្នកធ្វើការងារនៅកន្លែងសេវាកម្សាន្ត? | ................. ខែ<br>**ចំណាំ:**<br>ដាក់លេខ**១**ប្រសិនបើតិចជាងមួយខែ | | |
| 24 | តើដំបូងបំផុតអ្នកធ្វើការនៅសេវាកម្សាន្តប្រភេទណា? | ខារ៉ាអូខេ<br>ហាងម៉ាស្សា<br>បៀរហ្គាឌិន<br>ភោជនីយដ្ឋាន/ហាងកាហ្វេ<br>ក្លឹបរាំកម្សាន្ត<br>ឯករាជ្យ (តាមដងផ្លូវ សួនច្បារសាធារណៈ)<br>ផ្សេងៗសូមបញ្ជាក់--------------- | 1<br>2<br>3<br>4<br>5<br>6<br>7 | |
| 25 | តើពេលនេះអ្នកធ្វើការនៅសេវាកម្សាន្តប្រភេទណា? | ខារ៉ាអូខេ<br>ហាងម៉ាស្សា<br>បៀរហ្គាឌិន<br>ភោជនីយដ្ឋាន/ហាងកាហ្វេ<br>ក្លឹបរាំកម្សាន្ត<br>ឯករាជ្យ (តាមដងផ្លូវ សួនច្បារសាធារណៈ)<br>ផ្សេងៗសូមបញ្ជាក់--------------- | 1<br>2<br>3<br>4<br>5<br>6<br>7 | |
| 26 | តើជាទូទៅអ្នករកបានចំណូលពីការងារសេវាកម្សាន្តរបស់អ្នកប៉ុន្មានក្នុង១ខែ? | ..........................ដុល្លារអាមេរិក | | |
| 27 | តើអ្នកជាសមាជិកក្នុងអង្គការណាមួយដែលធ្វើការងារគាំទ្រដល់អ្នកបំរើសេវាកំសាន្តសប្បាយឬទេ? | ទេ<br>ចាស៎ | 0<br>1 | |
| **ផ្នែកទី ៣៖ ចំណេះដឹងពីសុខភាពផ្លូវភេទ អាកប្បកិរិយា និងឥរិយាបថ** | | | | |
| 28 | តើអ្នកបានរួមភេទឬទេក្នុងកំឡុងបីខែកន្លងមកនេះ? | ទេ<br>ចាស | 0<br>1 | 0<br>រំលងទៅសំណួរទី44 |
| 29 | តើអ្នកបានរួមភេទមិនដើម្បីប្តូរយកប្រាក់ឬរបស់របរ ជាមួយដៃគូ (មិត្តប្រុស/សង្សា) | ទេ<br>ចាស | 0<br>1 | 0<br>រំលងទៅសំណួរទី34 |

| | | | | |
|---|---|---|---|---|
| | ឬទេក្នុងកំឡុងបីខែកន្លងមកនេះ ? | | | |
| 30 | ក្នុងកំឡុងបីខែកន្លងមកនេះតើអ្នកបានរួមភេទមិនដើម្បីប្តូរយកប្រាក់ឬរបស់របរ ជាមួយដៃគូប៉ុន្មាននាក់? | ...........................នាក់ | | |
| 31 | ពេលចុងក្រោយដែលអ្នកបានរួមភេទមិនដើម្បីប្តូរយកប្រាក់ឬរបស់របរ ជាមួយដៃគូ (មិត្តប្រុស/សង្សា) តើដៃគូររបស់អ្នកបានប្រើប្រាស់ស្រោមអនាម័យឬទេ? | ទេ<br>ចាស | 0<br>1 | |
| 32 | ក្នុងកំឡុងបីខែកន្លងមក ពេលដែលអ្នកបានរួមភេទមិនដើម្បីប្តូរយកប្រាក់ឬរបស់របរ ជាមួយដៃគូ (មិត្តប្រុស/សង្សា) តើដៃគូរបស់អ្នកបានប្រើប្រាស់ស្រោមអនាម័យញឹកញាប់ប៉ុណ្ណា? | រាល់លើក<br>ញឹកញាប់<br>ពេលខ្លះ<br>មិនដែល | 1<br>2<br>3<br>4 | |
| 33 | ពេលចុងក្រោយដែលអ្នកបានរួមភេទមិនដើម្បីប្តូរយកប្រាក់ឬរបស់របរ ជាមួយដៃគូ (មិត្តប្រុស/សង្សា) ដោយមិនបានប្រើប្រាស់ស្រោមអនាម័យ តើអ្វីជាមូលហេតុចំបង? | ជឿទុកចិត្តដៃគូរ<br>មិនមានស្រោមអនាម័យ<br>ខ្ញុំបានស្នើសុំតែដៃគូខ្ញុំមិនចង់ប្រើ<br>ខ្ញុំបានស្នើសុំតែដៃគូខ្ញុំប្រាប់ថាមិនអីទេមិនបាច់ប្រើទេ<br>ខ្ញុំមិនបានស្នើសុំព្រោះតែខ្ញុំមានអារម្មណ៍មិនស្រួលឬក៏ខ្លាចក្នុងការស្នើសុំ<br>ខ្ញុំមិនបានស្នើសុំព្រោះតែខ្ញុំមិនបានគិតអំពីវា<br>ខ្ញុំមិនចូលចិត្តប្រើស្រោមអនាម័យទេ<br>ខ្ញុំខ្លាចថាប្រើស្រោមអនាម័យមានផលប៉ះពាល់ដល់ខ្លួនខ្ញុំ<br>ផ្សេងៗសូមបញ្ជាក់--------------- | 0<br>1<br>2<br>3<br>4<br>5<br>6<br>7<br>8 | |
| 34 | តើអ្នកបានរួមភេទដើម្បីប្តូរយកប្រាក់ឬរបស់របរ ជាមួយដៃគូ (ភ្ញៀវ) ឬទេក្នុងកំឡុងបីខែកន្លងមកនេះ ? | ទេ<br>ចាស់ | 0<br>1 | 0 រំលងទៅសំណួរទី44 |
| 35 | តើអ្វីដែលជាមូលហេតុចំបងធ្វើឲ្យអ្នករួមភេទជាមួយភ្ញៀវ? (ចម្លើយមានលើសពីមួយ) | ត្រូវការលុយ<br>មិត្តភក្តិបបួល<br>ត្រូវបានគេល្បួង ចាញ់បោកគេ ត្រូវគេបង្ខំ<br>ចាកចេញពីស្ថានភាពមិនល្អក្នុងគ្រួសារ | 1<br>2<br>3<br>4 | |

| | | | | |
|---|---|---|---|---|
| | | ចាកចេញពីការងារផ្សេងដែលអ្នកមិនចូលចិត្ត<br>ផ្សេងៗសូមបញ្ជាក់--------------- | 5<br>6 | |
| 36 | តើអ្នកមានអាយុប៉ុន្មានពេលដែលអ្នកចាប់ផ្តើមរួមភេទដើម្បីប្តូរយកប្រាក់ឬរបស់របរ ជាមួយដៃគូ (ភ្ញៀវ)លើកដំបូង? | -----------------ឆ្នាំ | | |
| 37 | ក្នុងកំឡុងបីខែមុនតើអ្នករួមភេទដើម្បីប្តូរយកប្រាក់ឬរបស់របរ ជាមួយដៃគូ (ភ្ញៀវ)ញឹកញាប់ប៉ុណ្ណា? | រាល់ថ្ងៃ<br>ពីរ បីដង ក្នុងមួយសប្តាហ៍<br>រាល់សប្តាហ៍<br>រាល់ខែ<br>ម្តងម្កាលពេលដែលខ្ញុំគិតថាចង់/ត្រូវការ | 1<br>2<br>3<br>4<br>5 | |
| 38 | តើភាគច្រើនអ្នកជួបភ្ញៀវនៅកន្លែងណា? | កន្លែងធ្វើការ<br>តាមរយៈមិត្តភក្តិ<br>តាមការជួបជុំកម្មវិធីសង្គម<br>តាមផ្លូវ<br>ការផ្សព្វផ្សាយ/ទូរស័ព្ទ<br>ផ្សេងៗសូមបញ្ជាក់--------------- | 1<br>2<br>3<br>4<br>5<br>6 | |
| 39 | ក្នុងកំឡុងបីខែកន្លងមកនេះតើអ្នកបានរួមភេទដើម្បីប្តូរយកប្រាក់ឬរបស់របរ ជាមួយដៃគូប៉ុន្មាននាក់? | ..........................នាក់ | | |
| 40 | ក្នុងកំឡុង**7**ថ្ងៃកន្លងមកនេះតើអ្នកបានរួមភេទដើម្បីប្តូរយកប្រាក់ឬរបស់របរ ជាមួយដៃគូប៉ុន្មាននាក់? | ..........................នាក់ | | |
| 41 | ពេលចុងក្រោយដែលអ្នកបានរួមភេទដើម្បីប្តូរយកប្រាក់ឬរបស់របរ ជាមួយដៃគូ (ភ្ញៀវ)<br>តើដៃគូរបស់អ្នកបានប្រើប្រាស់ស្រោមអនាម័យឬទេ? | ទេ<br>ចាស | 0<br>1 | |
| 42 | ក្នុងកំឡុងបីខែកន្លងមកនេះ ពេលដែលអ្នកបានរួមភេទដើម្បីប្តូរយកប្រាក់ឬរបស់របរ ជាមួយដៃគូ (ភ្ញៀវ)<br>តើដៃគូរបស់អ្នកបានប្រើប្រាស់ស្រោមអនាម័យញឹកញាប់ប៉ុណ្ណា? | ជានិច្ច<br>ញឹកញាប់<br>ម្តងម្កាល<br>មិនដែល | 1<br>2<br>3<br>4 | |
| 43 | ពេលចុងក្រោយដែលអ្នកបានរួមភេទដើម្បីប្តូរយកប្រាក់ឬរបស់របរ ជាមួយដៃគូ (ភ្ញៀវ)<br>ដោយមិនបានប្រើប្រាស់ស្រោមអនាម័យ តើអ្វីជាមូលហេតុចំបង? | ជឿទុកចិត្តដៃគូរ<br>មិនមានស្រោមអនាម័យ<br>ខ្ញុំបានស្នើសុំតែដៃគូខ្ញុំមិនចង់<br>ប្រើ<br>ខ្ញុំបានស្នើសុំតែដៃគូខ្ញុំប្រាប់ថាមិនអីទេមិនបាច់ប្រើទេ | 0<br>1<br>2<br>3<br><br>4 | |

| | | | | |
|---|---|---|---|---|
| | | ខ្ញុំមិនបានស្នើសុំព្រោះតែខ្ញុំមានអារម្មណ៍មិនស្រួលឬក៏ខ្លាចក្នុងការស្នើសុំ<br>ខ្ញុំមិនបានស្នើសុំព្រោះតែខ្ញុំមិនបានគិតអំពីវា<br>ខ្ញុំមិនចូលចិត្តប្រើស្រោមអនាម័យទេ<br>ខ្ញុំខ្លាចថាប្រើស្រោមអនាម័យមានផលប៉ះពាល់ដល់ខ្លួនខ្ញុំ<br>ផ្សេងៗសូមបញ្ជាក់--------------- | 5<br>6<br>7<br>8 | |
| **ផ្នែកទី ៤៖ ប្រសិទ្ធភាពនៃការប្រើប្រាស់ស្រោមអនាម័យដោយខ្លួនឯង** | | | | |
| 44 | តើអ្នកអាចចចារធ្វើឲ្យដៃគូរួមភេទរបស់អ្នកប្រើស្រោមអនាម័យបានដែរឬទេ? | ពិតជាមិនអាច<br>ប្រហែលអាច<br>ពិតជាអាច | 0<br>1<br>2 | |
| 45 | តើអ្នកអាចនៅតែទទូចចង់ប្រើស្រោមអនាម័យដែរឬទេគ្រប់ពេលដែលដៃគូរបស់អ្នកនិយាយថាគាត់មិនមានផ្ទុកជម្ងឺអ្វីទាំងអស់? | ពិតជាមិនអាច<br>ប្រហែលអាច<br>ពិតជាអាច | 0<br>1<br>2 | |
| 46 | តើអ្នកនឹងសុំឲ្យដៃគូអ្នកប្រើស្រោមដែរឬទេនៅពេលដែលអ្នកមិនមែនជាអ្នកចាប់ផ្តើមសកម្មភាពផ្លូវភេទ? | ពិតជាមិនអាច<br>ប្រហែលអាច<br>ពិតជាអាច | 0<br>1<br>2 | |
| 47 | តើអ្នកអាចនៅតែទទូចចង់ប្រើស្រោមអនាម័យដែរឬទេប្រសិនបើអតិថិជនមិនចង់ប្រើវា? | ពិតជាមិនអាច<br>ប្រហែលអាច<br>ពិតជាអាច | 0<br>1<br>2 | |
| 48 | តើអ្នកនឹងបញ្ឈប់ការរួមភេទដែរឬទេបើដៃគូរបស់អ្នកនៅតែទទូចមិនចង់ប្រើស្រោមអនាម័យ? | ពិតជាមិនអាច<br>ប្រហែលអាច<br>ពិតជាអាច | 0<br>1<br>2 | |
| 49 | តើអ្នកនៅតែបន្តទទូចសុំអោយប្រើស្រោមអនាម័យដែរឬទេបើសិនជាដៃគូអ្នកបានខឹងនៅពេលដែលអ្នកសុំឲ្យគេប្រើស្រោម? | ពិតជាមិនអាច<br>ប្រហែលអាច<br>ពិតជាអាច | 0<br>1<br>2 | |
| **ផ្នែកទី ៥៖ កត្តាប្រឈមមេរោគអេដស៍ ការធ្វើតេស្តឈាម និងការព្យាបាល** | | | | |
| 50 | តើអ្នកគិតថាអ្នកប្រឈមមុខទៅនឹងការឆ្លងមេរោគអេដស៍ដែរឬទេ? | ទេ<br>ចាស<br>មិនប្រាកដ | 0<br>1<br>2 | 0<br>រំលងទៅសំណួរទី52 |
| 51 | បើសិនជាចំលើយចាស ហេតុអ្វីបានជាអ្នកគិតថាអ្នកប្រឈមមុខទៅនឹងការឆ្លងមេរោគអេដស៍? | ខ្ញុំដឹងថាដៃគូររបស់ខ្ញុំមានផ្ទុកមេរោគអេដស៍<br>ខ្ញុំតែងតែរួមភេទមិនបានការពារជាញឹកញាប់<br>ខ្ញុំមិនទុកចិត្តប្ដី រឺដៃគូរបស់ខ្ញុំ | 1<br>2<br>3 | |

| | | | | |
|---|---|---|---|---|
| | | ខ្ញុំបានប្រើម្ជុលរួមគ្នាក្នុងការចាក់គ្រឿងញៀន<br>ខ្ញុំមានសមាជិកគ្រួសារដែលមានផ្ទុកមេរោគអេដស៍<br>ខ្ញុំជាអ្នកថែទាំអ្នកផ្ទុកមេរោគអេដស៍<br>ផ្សេងៗសូមបញ្ជាក់--------------- | 4<br>5<br>6<br>7 | |
| 52 | បើសិនជាចំលើយទេ ហេតុអ្វីបានជាអ្នកមិនគិតថាអ្នកប្រឈមមុខទៅនឹងការឆ្លងមេរោគអេដស៍? | មិនដែលរួមភេទ/មិនមានដៃគូរ<br>ខ្ញុំរួមភេទតែជាមួយប្តីរបស់ខ្ញុំ<br>ខ្ញុំមិនគិតថាដៃគូរបស់ខ្ញុំមានផ្ទុកមេរោគអេដស៍<br>ខ្ញុំគ្មានអារម្មណ៍ថាមានជម្ងឺ<br>ខ្ញុំតែងតែប្រើស្រោមអនាម័យខ្ញុំតែងតែធ្វើតេស្តរាល់ ៣-៦ខែ<br>ខ្ញុំតែងតែលាងសំអាតបន្ទាប់ពីរួមភេទ<br>ខ្ញុំតែងតែទៅសំអាតនៅគ្លីនិកជាញឹកញាប់<br>ផ្សេងៗសូមបញ្ជាក់--------------- | 0<br>1<br>2<br>3<br>4<br>5<br>6<br>7<br>8 | |
| 53 | តើអ្នកធ្លាប់បានធ្វើតេស្តរកមេរោគអេដស៍ដែររឺទេ? | ទេ<br>ចាស | 0<br>1 | 0 រំលងទៅសំណួរទី61 |
| 54 | ក្នុងកំឡុង៦ខែមុនតើអ្នកធ្លាប់បានធ្វើតេស្តរកមេរោគអេដស៍ដែររឺទេ? | ទេ<br>ចាស | 0<br>1 | 0 រំលងទៅសំណួរទី61 |
| 55 | តើអ្នកធ្វើតេស្តរកមេរោគអេដស៍ចុងក្រោយនៅឯណា? | មន្ទីរពេទ្យឯកជន<br>មន្ទីរពេទ្យរដ្ឋ<br>មន្ទីរពេទ្យអង្គការ<br>នៅកន្លែងធ្វើការឬផ្ទះរបស់បុគ្គលិកសហគមន៍របស់អង្គការ<br>ផ្សេងៗសូមបញ្ជាក់------- | 1<br>2<br>3<br>4<br>5<br>6 | |
| 56 | ប្រសិនបើមិនប្រកាន់ សូមប្រាប់ខ្ញុំពីលទ្ធផលតេស្តឈាមរបស់អ្នក (អ្នកមានសិទ្ធក្នុងការមិនឆ្លើយ) | មិនមានផ្ទុក<br>មានផ្ទុក<br>មិនដឹង<br>មិនចង់ឆ្លើយ | 1<br>2<br>3<br>4 | 1 និង4រំលងទៅសំណួរ61 |
| 57 | តើអ្នកកំពុងតែប្រើថ្នាំពន្យាជីវិត(ART) ឬទេ? | ទេ<br>ចាស<br>ឈប់ប្រើ | 0<br>1<br>2 | 0 2 3 រំលងទៅសំណួរទី60 |

| | | | | |
|---|---|---|---|---|
| | | សេវាមុនទទួលថ្នាំពន្យារ | 3 | |
| 58 | តើអ្នកបានលេបថ្នាំពន្យាជីវិត(ART ) ទៀងទាត់ តាមវេជ្ជបញ្ជាឬទេ? | មិនទៀងទាត់<br>ទៀងទាត់ | 0<br>1 | |
| 59 | តើអ្នកទទួលសេវាART នៅទីណា? | មន្ទីរពេទ្យរដ្ឋ<br>មន្ទីរពេទ្យឯកជន<br>មន្ទីរពេទ្យអង្គការ<br>ឱសថស្ថាន<br>ផ្សេងៗសូមបញ្ជាក់--------------- | 1<br>2<br>3<br>4<br>5 | ចម្លើយទាំងអស់រំលងទៅសំណួរ61 |
| 60 | ប្រសិនបើទេ ហេតុអ្វីបានជាមិនប្រើប្រាស់ថ្នាំពន្យា? | មិនត្រូវការ<br>មិនច្បាស់ថាត្រូវទៅរកឯណា<br>ខ្មាសមិនហ៊ានទៅរកសេវាសុខភាព<br>ខ្លាចត្រូវគេរើសអើង<br>ធ្លាប់មានបទពិសោធន៍មិនល្អនៅកន្លែងផ្តល់សេវា<br>ផ្សេងៗសូមបញ្ជាក់--------------- | 1<br>2<br>3<br>4<br>5<br>6 | |

## ផ្នែកទី ៦៖ ការធ្វើតេស្ត និងព្យាបាលជំងឺកាមរោគ

| | | | | |
|---|---|---|---|---|
| 61 | បីខែកន្លងមកតើអ្នកធ្លាប់កើតមាននូវរោគសញ្ញាទាំងនេះដែររឺទេ? (ចម្លើយមានលើសពីមួយ) | គ្មានរោគសញ្ញា<br>ដាច់ រឺឈឺនៅតំបន់ប្រដាប់ភេទ<br>ហើមនៅតំបន់ប្រដាប់ភេទ<br>ធ្លាក់សមានក្លិនមិនល្អ រមាស់<br>រឺលាកនៅតំបន់ប្រដាប់ភេទ<br>មានរោគសញ្ញានៅមាត់/បំពង់ក<br>ផ្សេងៗសូមបញ្ជាក់------- | 0<br>1<br>2<br>3<br>4<br>5 | 0 រំលងទៅសំណួរទី66 |
| 62 | តើអ្នកបានធ្វើតេស្ត រកជំងឺកាមរោគឬ ទេពេលដែលអ្នកមានសញ្ញាទាំងនោះ? | ទេ<br>ចាស | 0<br>1 | 0 រំលងទៅសំណួរទី66 |
| 63 | កាលពីពេល៦ខែកន្លងមកនេះតើគ្រូពេទ្យធ្លាប់ប្រាប់ថាអ្នកមានជំងឺកាមរោគឬទេ? | ទេ<br>ចាស | 0<br>1 | 0 រំលងទៅសំណួរទី66 |
| 64 | តើអ្នកមានទទួលការព្យាបាលជំងឺកាមរោគចុងក្រោយរឺទេ? | ទេ<br>ចាស | 0<br>1 | 0 រំលងទៅសំណួរទី66 |
| 65 | តើអ្នកទទួលការព្យាបាលនៅកន្លែងណា? | ឱសថស្ថាន<br>គ្លីនិកឬមន្ទីរពេទ្យឯកជន<br>គ្លីនិកឬមន្ទីរពេទ្យរដ្ឋ | 0<br>1<br>2<br>3<br>4 | |

| | | | | |
|---|---|---|---|---|
| | | គ្លីនិកឬមន្ទីរពេទ្យអង្គការ<br>ការព្យាបាលតាមបែបបុរាណ<br>ផ្សេងៗសូមបញ្ជាក់--------------- | 5 | |
| **ផ្នែកទី ៧៖ ការពន្យារកំណើតនិងការមានផ្ទៃពោះ** | | | | |
| 66 | តើអ្នកកំពុងប្រើប្រាស់មធ្យោបាយពន្យារកំណើតទំនើបដើម្បីបង្ការការមានផ្ទៃពោះដែររឺទេ? | ទេ<br>ចាស | 0<br>1 | 0<br>រំលងទៅសំណួរទី68 |
| 67 | បើសិនជាអ្នកកំពុងប្រើតើអ្នកប្រើប្រភេទអ្វី?<br>(ចម្លើយអាចលើសពីមួយ) | ស្រោមអនាម័យបុរស<br>ថ្នាំគ្រាប់<br>ថ្នាំចាក់<br>កងក្នុងស្បូន<br>កងដាក់ក្រោមស្បែក<br>ការចងដៃស្បូន<br>ចងបំពង់មេជីវិតឈ្មោល<br>ផ្សេងៗសូមបញ្ជាក់--------------- | 1<br>2<br>3<br>4<br>5<br>6<br>7<br>8 | ចម្លើយទាំងអស់រំលងទៅសំណួរ69 |
| 68 | បើសិនជាមិនកំពុងប្រើប្រាស់ទេ ហេតុអ្វីបានជាមិនប្រើមធ្យោបាយពន្យារកំណើតទំនើប? | មិនចូលចិត្តផលរំខានរបស់វា<br>គិតថាមិនត្រូវការការពន្យាកំណើត<br>មិនចង់ពន្យារកំណើត/ចង់មានកូន<br>មិនចូលចិត្តប្រើមធ្យោបាយទំនើប<br>មិនដឹងទៅស្វែងរកមធ្យោបាយពន្យារកំនើតនៅទីណា<br>មិនគិតថាខ្ញុំអាចមានលទ្ធភាពប្រើមធ្យោបាយពន្យារកំនើត<br>ផ្សេងៗសូមបញ្ជាក់----------------- | 1<br>2<br>3<br>4<br>5<br>6<br>7 | |
| 69 | តើអ្នកធ្លាប់លាងសម្អាតប្រដាប់ភេទក្រោយរួមភេទដើម្បីបង្ការមេរោគអេដស៍ រឺជំងឺកាមរោគដែររឺទេ? | ទេ<br>ចាស | 0<br>1 | 0<br>រំលងទៅសំណួរ72 |
| 70 | បើសិនជាធ្លាប់<br>តើអ្នកលាងសម្អាតខ្លួនក្រោយរួមភេទដើម្បីបង្ការមេរោគអេដស៍ រឺកាមរោគញឹកញាប់ប៉ុណ្ណា? | ជានិច្ច<br>ញឹកញាប់<br>ម្តងម្កាល<br>មិនដែល | 1<br>2<br>3<br>4 | |
| 71 | បើសិនជាអ្នកលាងសំអាតក្រោយពេលរួមភេទ<br>តើអ្វីខ្លះដែលជាហេតុផលក្នុងការលាងសម្អាតប្រដាប់ភេទរបស់អ្នក? | បង្ការជម្ងឺកាមរោគ<br>បង្ការជម្ងឺអេដស៍<br>បង្ការផ្ទៃពោះ<br>ធ្វើឲ្យស្អាត<br>ជៀសវាងក្លិនមិនល្អ<br>ផ្សេងៗសូមបញ្ជាក់--------------- | 1<br>2<br>3<br>4<br>5<br>6 | |

| | | | | |
|---|---|---|---|---|
| | (ចម្លើយអាចលើសពីមួយ) | | | |
| 72 | តើអ្នកគិតថាការរំលូតកូន(មុន១២អាទិត្យនៃការបង្កកំណើត) ស្របច្បាប់ក្នុងប្រទេសកម្ពុជាដែររឺទេ? | ទេ<br>ចាស<br>មិនដឹង | 0<br>1<br>2 | |
| 73 | តើអ្នកធ្លាប់មានផ្ទៃពោះដោយចៃដន្យដែរឬទេ? | ទេ<br>ចាស<br>មិនដឹង | 0<br>1<br>2 | 0<br>រំលងទៅ<br>សំណួរ82 |
| 74 | តើអ្នកធ្លាប់មានផ្ទៃពោះដោយចៃដន្យដែរឬទេក្នុងកំឡុង១២ខែកន្លងមក? | ទេ<br>ចាស<br>មិនដឹង | 0<br>1<br>2 | |
| 75 | តើអ្នកធ្លាប់រំលូតកូនឬទេ? | ទេ<br>ចាស<br>មិនដឹង | 0<br>1<br>2 | 0<br>រំលងទៅ<br>សំណួរ82 |
| 76 | គិតមកដល់ពេលនេះ តើអ្នកធ្លាប់រំលូតកូនប៉ុន្មានដង? | ………………………ដង | | |
| 77 | ក្នុងកំឡុង១២ខែមុន តើអ្នកធ្លាប់រំលូតកូនប៉ុន្មានដង? | ………………………ដង | | |
| 78 | តើអ្នកប្រើប្រាស់សេវាណាសំរាប់ការរំលូតកូនលើកចុងក្រោយ? | ឱសថស្ថាន<br>គ្លីនិកឬមន្ទីរពេទ្យឯកជន<br>គ្លីនិកឬមន្ទីរពេទ្យរដ្ឋ<br>គ្លីនិកឬមន្ទីរពេទ្យអង្គការ<br>ថ្នាំបុរាណ<br>ផ្សេងៗសូមបញ្ជាក់--------------- | 0<br>1<br>2<br>3<br>4<br>5 | |
| 79 | តើអ្នកធ្លាប់មានផលវិបាកបន្ទាប់ពីការរំលូតដែររឺទេ?(ដូចជាការហូរឈាមច្រើនរឺការបង្ករោគ) | ទេ<br>ចាស | 0<br>1 | 0<br>រំលងទៅ<br>សំណួរ82 |
| 80 | បើសិនជាធ្លាប់ តើអ្នកបានស្វែងរកការព្យាបាលសម្រាប់ផលវិបាកទាំងអស់នេះដែររឺទេ? | ទេ<br>ចាស | 0<br>1 | 0<br>រំលងទៅ<br>សំណួរ82 |
| 81 | បើធ្លាប់តើអ្នកស្វែងរកការព្យាបាលនៅទីណា?<br>(ចម្លើយអាចលើសពីមួយ) | ឱសថស្ថាន<br>គ្លីនិកឬមន្ទីរពេទ្យឯកជន<br>គ្លីនិកឬមន្ទីរពេទ្យរដ្ឋ<br>គ្លីនិកឬមន្ទីរពេទ្យអង្គការ<br>ថ្នាំបុរាណ<br>ផ្សេងៗសូមបញ្ជាក់--------------- | 0<br>1<br>2<br>3<br>4<br>5 | |

| ផ្នែកទី ៨: អំពើហឹង្សាផ្អែកលើយេនឌ័រ | | | | |
|---|---|---|---|---|
| 82 | តើអ្នកធ្លាប់ត្រូវបានគេប៉ះពាល់ដោយអ្នកមិនចង់នៅក្នុងរយ:ពេល៦ខែចុងក្រោយនេះឬទេ? | ទេ<br>ចាស | 0<br>1 | |
| 83 | តើអ្នកអាចធ្វើអ្វីបាន បើសិនជាអ្នក រឺមិត្តភក្តិស្រីរបស់អ្នក រឺសមាជិកគ្រួសារអ្នកត្រូវបានគេបំពាន?<br>(ចម្លើយអាចលើសពីមួយ) | មិនមានអ្វីដែលត្រូវធ្វើ<br>អាជ្ញាធរមូលដ្ឋាន<br>ទៅរកប៉ូលីស រឺតុលាការ<br>ប្រាប់សមាជិកគ្រួសារ និងមិត្តភក្តិ<br>ទៅរកអង្គការ (NGO)<br>ផ្សេងៗសូមបញ្ជាក់--------------- | 1<br>2<br>3<br>4<br>5<br>6 | |
| 84 | ប្រសិនបើប្រពន្ធមិនគោរពប្តី តើអ្នកគិតថាប្តីវ៉ៃ ទាត់ រឺបំពានប្រពន្ធជាការសមហេតុផលដែររឺទេ? | ទេ<br>ចាស់<br>ពេលខ្លះ | 0<br>1<br>2 | |
| 85 | ប្រសិនបើប្រពន្ធមិនគោរពប្តី តើអ្នកគិតថាសមហេតុផលដែររឺទេដែលប្តីស្រែកឲ្យប្រពន្ធ? | ទេ<br>ចាស<br>ពេលខ្លះ | 0<br>1<br>2 | |
| 86 | ប្រសិនបើមិត្តស្រីមិនគោរពមិត្តប្រុស តើអ្នកគិតថាមិត្តប្រុសមានហេតុផលក្នុងការបំពានរាងកាយនាងដែររឺទេ? | ទេ<br>ចាស<br>ពេលខ្លះ | 0<br>1<br>2 | |
| 87 | តើអ្នកគិតថាវាជាតួនាទីស្ត្រីក្នុងការរួមភេទជាមួយប្តីបើទោះបីជាស្ត្រីមិនចង់ក៍ដោយ? | ទេ<br>ចាស<br>ពេលខ្លះ | 0<br>1<br>2 | |
| 88 | តើអំពើហិង្សាប្រភេទណា ដែលអ្នកធ្លាប់ជួបក្នុងជិវិតរបស់អ្នក?<br>(ចម្លើយអាចលើសពីមួយ) | មិនធ្លាប់<br>ការគំរាមដោយពាក្យសម្តី<br>ហាមមិនឲ្យចេញក្រៅផ្ទះ<br>អំពើហិង្សាលើរាងកាយ<br>បង្ខំឲ្យរួមភេទ<br>បង្ខំឲ្យប្រើគ្រឿងស្រវឹង<br>បង្ខំឲ្យប្រើគ្រឿងញៀន<br>ផ្សេងៗសូមបញ្ជាក់--------------- | 0<br>1<br>2<br>3<br>4<br>5<br>6<br>7 | 0<br>រំលងទៅសំណួរ91 |
| 89 | តើអំពើហិង្សាប្រភេទណាដែលអ្នកធ្លាប់ជួបនៅ៦ខែចុងក្រោយនេះ? | មិនធ្លាប់<br>ការគំរាមដោយពាក្យសម្តី<br>ហាមមិនឲ្យចេញក្រៅផ្ទះ<br>អំពើហិង្សាលើរាងកាយ<br>បង្ខំឲ្យរួមភេទ<br>បង្ខំឲ្យប្រើគ្រឿងស្រវឹង<br>បង្ខំឲ្យប្រើគ្រឿងញៀន<br>ផ្សេងៗសូមបញ្ជាក់--------------- | 1<br>2<br>3<br>4<br>5<br>6<br>7<br>8 | 1<br>រំលងទៅសំណួរ 91 |

| | | | | |
|---|---|---|---|---|
| 90 | អ្នកណាជាអ្នកប្រព្រឹត្តអំពើហឹង្សាជាចំបងមកលើអ្នក?<br>(ចម្លើយអាចលើសពីមួយ) | សមាជិកគ្រួសារ<br>មិត្តភក្តិ<br>ប្តី/ដៃគូ<br>សង្សា<br>មេកា/អ្នកគ្រប់គ្រងហាង<br>ភ្ញៀវ<br>អ្នករួមការងារ<br>ជនមិនស្គាល់<br>អាជ្ញាធរមូលដ្ឋាន/ប៉ូលីស<br>អ្នករត់ម៉ូតូឌុប/អ្នកបើកឡានតាក់ស៊ី<br>ផ្សេងៗសូមបញ្ជាក់--------------- | 1<br>2<br>3<br>4<br>5<br>6<br>7<br>8<br>9<br>10<br>11 | |
| **ផ្នែកទី ៩៖ ការប្រើប្រាស់សារធាតុញៀន** | | | | |
| 91 | តើអ្នកផឹកស្រាបៀយ៉ាងហោចណាស់មួយកំប៉ុងឬស្រាមួយកែវប៉ុន្មានដងក្នុងកំឡុង៣ខែមុន?<br><br>ស្រាមួយកែវ ១២០ មល<br>វីស្គីមួយកែវ ៣០ មល | មិនដែល<br>មួយខែម្តង<br>2-4 ដងក្នុងមួយខែ<br>2-3 ដងក្នុងមួយសប្តាហ៍<br>4 ឬច្រើនដងក្នុងមួយសប្តាហ៍ | 0<br>1<br>2<br>3<br>4 | 0<br>រំលងទៅសំណួរ94 |
| 92 | ជាធម្មតាតើអ្នកផឹកស្រាបៀ ឬស្រាប៉ុន្មានកំប៉ុង/កែវ ក្នុងមួយថ្ងៃ?<br>(គិតតាមស្តង់ដាសំណួរទី91) | 1-2<br>3-4<br>5-6<br>7-9<br>10 ឬច្រើនជាង | 0<br>1<br>2<br>3<br>4 | |
| 93 | ក្នុងកំឡុង៣ខែកន្លងមកតើអ្នកផឹកគ្រឿងស្រវឹងច្រើនជាង៤កែវ/កំប៉ុងក្នុង២៤ម៉ោង ញឹកញាប់ប៉ុណ្ណា? | មិនដែល<br>តិចជាងម្តងក្នុងមួយខែ<br>ម្តងក្នុងមួយខែ<br>1, 2 ឬ 3 ដងក្នុងមួយសប្តាហ៍<br>4 ឬច្រើនដងក្នុងមួយសប្តាហ៍ | 0<br>1<br>2<br>3<br>4 | |
| 94 | តើអ្នកត្រូវបានបង្ខំអោយផឹកគ្រឿងស្រវឹងនៅន្លែងធ្វើការរបស់អ្នកញឹកញាប់ប៉ុណ្ណាក្នុងកំឡុង3ខែកន្លងមក? | មិនដែល<br>តិចជាងម្តងក្នុងមួយខែ<br>ម្តងក្នុងមួយខែ<br>1, 2 ឬ 3 ដងក្នុងមួយសប្តាហ៍<br>4 ឬច្រើនដងក្នុងមួយសប្តាហ៍ | 0<br>1<br>2<br>3<br>4 | |
| 95 | ក្នុងកំឡុងបីខែកន្លងមកតើអ្នកមានបានប្រើថ្នាំញៀនទាំងនេះទេ? | មិនដែល<br>កញ្ឆា<br>អាភៀន/ហេរ៉ូអ៊ីន<br>យ៉ាម៉ា(អំហ្វេតាមីន)<br>ម៉ាទឹកកក<br>អ៊ិចស្តាស៊ី<br>ការហិត | 0<br>1<br>2<br>3<br>4<br>5<br>6 | 0 រំលងទៅសំណួរ Q98 |

| | | | | |
|---|---|---|---|---|
| | | ផ្សេងៗ(សូមបញ្ជាក់) | 7 | |
| 96 | ក្នុងកំឡុងបីខែកន្លងមកតើអ្នកមានបានប្រើប្រាស់ថ្នាំញៀនប្រភេទចាក់ឬទេ? | មិនដែលចាក់ថ្នាំញៀនទេ<br>ហេរ៉ូអ៊ីន<br>យ៉ាម៉ា<br>ម៉ាទឹកកក<br>ផ្សេងៗសូមបញ្ជាក់--------------- | 0<br>1<br>2<br>3<br>4 | |
| 97 | ក្នុងកំឡុងបីខែកន្លងមកតើអ្នកធ្លាប់បានរួមភេទកំឡុងពេលប្រើប្រាស់ថ្នាំញៀនឬទេ? | ទេ<br>ចាស់<br>មិនឆ្លើយ | 0<br>1<br>2 | |
| **ផ្នែកទី ១០៖ តំណរភ្ជាប់ទៅការទទួលសេវា** | | | | |
| 98 | តើអ្នកធ្លាប់ទាក់ទងទៅកាន់បុគ្គលិកសហគមន៍ដើម្បីសួរពីបញ្ហាសុខភាពដែររឺទេ? | ទេ<br>ចាស | 0<br>1 | 0<br>សូមបញ្ចប់ការសម្ភាស |
| 99 | តើបញ្ហាសុខភាពអ្វីដែលអ្នកទាក់ទងទៅកាន់ពួកគេ?<br>(ចម្លើយអាចលើសពីមួយ) | មេរោគអេដស៍/ជំងឺអេដស៍<br>ជំងឺកាមរោគ<br>(រួមទាំងស្វាយ)<br>ផែនការគ្រួសារ<br>អំពើហិង្សា<br>ការគាំទ្រផ្លូវចិត្តទូទៅ<br>សុខភាពទ្វាមាស(ធ្លាក់សរលាក ក្រហាយ)<br>ការគាំទ្រផ្នែកផ្លូវច្បាប់<br>ផ្សេងៗសូមបញ្ជាក់--------------- | 1<br>2<br>3<br>4<br>5<br>6<br><br>7<br>8 | |
| 100 | តើអ្នកទាក់ទងទៅកាន់បុគ្គលិកសហគមន៍ប៉ុន្មានដងក្នុងរយៈពេល៦ខែកន្លងមក? | មិនដែលទាក់ទង<br>១ ដង<br>២-៤ ដង<br>៥+ ដង | 0<br>1<br>2<br>3 | |
| 101 | តើអ្នកធ្លាប់ទទួលបានការបញ្ជូនទៅរកសេវាសុខភាពពីអ្នកបុគ្គលិកសហគមន៍ដែររឺទេ? | ទេ<br>ចាស | 0<br>1 | 0<br>សូមបញ្ចប់ការសម្ភាស |
| 102 | តើអ្នកត្រូវបានបញ្ជូនដោយសារមានបញ្ហាសុខភាពអ្វីដែរ?<br>(ចម្លើយមានលើសពីមួយ) | មេរោគអេដស៍/ជំងឺអេដស៍ជំងឺកាមរោគ<br>(រួមទាំងស្វាយ)<br>ផែនការគ្រួសារ<br>អំពើហិង្សា<br>ការគាំទ្រផ្លូវចិត្តទូទៅ<br>សុខភាពទ្វាមាស(ធ្លាក់សរលាក ក្រហាយ)<br>ការគាំទ្រផ្នែកផ្លូវច្បាប់<br>ផ្សេងៗសូមបញ្ជាក់--------------- | 1<br>2<br>3<br>4<br>5<br>6<br><br>7<br>8 | |
